# Report on the Nutritive Properties of Gelatine

**Published:** 1842-05-07

**Authors:** 


*Report of the French Academy on the Nutritive Properties of Gelatine.*—The committee, consisting of MM. Thenard, D'Arcet, Dumas, Flourens, Serres, Breschet, and Magendie, fed a number of dogs with gelatine, in its various states, and as obtained from various substances. They found that these animals, in general, obstinately refused to take it above once or twice, and died as speedily as if they had been completely deprived of food. Even when the gelatine was flavoured, by being boiled with a little fresh meat, though the dogs partook of it more readily at first, all died in the time they would have done, had they been deprived altogether of nourishment. When fed on a mixed diet, the dogs were perfectly nourished, and even when fed for months on raw bones, maintained their health and vigour unimpaired. Bones which had been boiled had not, however, the same nourishing qualities; the animals rapidly fell off when kept on that diet. The conclusions at which the committee arrived were the following:—

1. No known process can extract from bones an aliment which can supply the place of fresh meat.

2. Gelatine, albumen, or fibrine, taken separately, only nourish an animal for a very limited period, and in a very imperfect manner. In general, these substances excite in a short time an insurmountable disgust, so that the animals will rather die of hunger than eat them.

3. These same principles, artificially united, and rendered savoury by proper seasoning, are taken more freely and for a longer time than when separate, but, in the long run, they nourish the body no better; for the animals which eat them, even in considerable quantities, end by dying with all the signs of complete starvation.

4. Muscular flesh, in which gelatine, albumen, and fibrine are united according to the laws of nature, and are associated with other matters, as fat, salts, &c. suffice, even in small quantity, for complete and prolonged nutrition.

5. Raw bones have the same property, but the quantity consumed in twenty-four hours requires to be considerably greater than if fresh meat were given.

6. All kind of preparation, as decoction in water, the action of hydrochloric acid, and, above all, its transformation into gelatine, diminishes the nutritive quality of bones, and seems even in certain cases to deprive them of it altogether.

7. The committee do not take on them at present to decide as to the question of the nutritive powers of gelatine for human food when given along with other alimentary matters; they are aware that direct experiments on a large scale can only decide the question. They are engaged in this investigation at present, and will make known the results soon.

8. Gluten, as procured from the flour of wheat or of maize, suffices of itself for a complete and prolonged nutrition.

9. Fat substances, taken as the only aliment, support life for a long time, but they give rise to an imperfect and disordered nutrition, where fat accumulates in all the tissues, sometimes in the state of oleine and of stearine, sometimes in the state of almost pure stearine.—*Ed. Med. and Surg. Jour.*, from *Comptes Rendus des Seances de l'Academie des Sciences.* August, 1841.

